# Inhibitors of the Sec61 Complex and Novel High Throughput Screening Strategies to Target the Protein Translocation Pathway

**DOI:** 10.3390/ijms222112007

**Published:** 2021-11-05

**Authors:** Eva Pauwels, Ralf Schülein, Kurt Vermeire

**Affiliations:** 1 KU Leuven Department of Microbiology, Immunology and Transplantation, Rega Institute for Medical Research, Laboratory of Virology and Chemotherapy, B-3000 Leuven, Belgium; eva.pauwels@kuleuven.be; 2 Leibniz-Forschungsinstitut für Molekulare Pharmakologie, Robert-Rössle-Str. 10, 13125 Berlin, Germany; Schuelein@fmp-berlin.de

**Keywords:** signal recognition particle dependent protein targeting, Sec61 dependent translocation, co-translational translocation, endoplasmic reticulum, inhibitor, high throughput screening

## Abstract

Proteins targeted to the secretory pathway start their intracellular journey by being transported across biological membranes such as the endoplasmic reticulum (ER). A central component in this protein translocation process across the ER is the Sec61 translocon complex, which is only intracellularly expressed and does not have any enzymatic activity. In addition, Sec61 translocon complexes are difficult to purify and to reconstitute. Screening for small molecule inhibitors impairing its function has thus been notoriously difficult. However, such translocation inhibitors may not only be valuable tools for cell biology, but may also represent novel anticancer drugs, given that cancer cells heavily depend on efficient protein translocation into the ER to support their fast growth. In this review, different inhibitors of protein translocation will be discussed, and their specific mode of action will be compared. In addition, recently published screening strategies for small molecule inhibitors targeting the whole SRP-Sec61 targeting/translocation pathway will be summarized. Of note, slightly modified assays may be used in the future to screen for substances affecting SecYEG, the bacterial ortholog of the Sec61 complex, in order to identify novel antibiotic drugs.

## 1. Introduction

**Figure 1:**
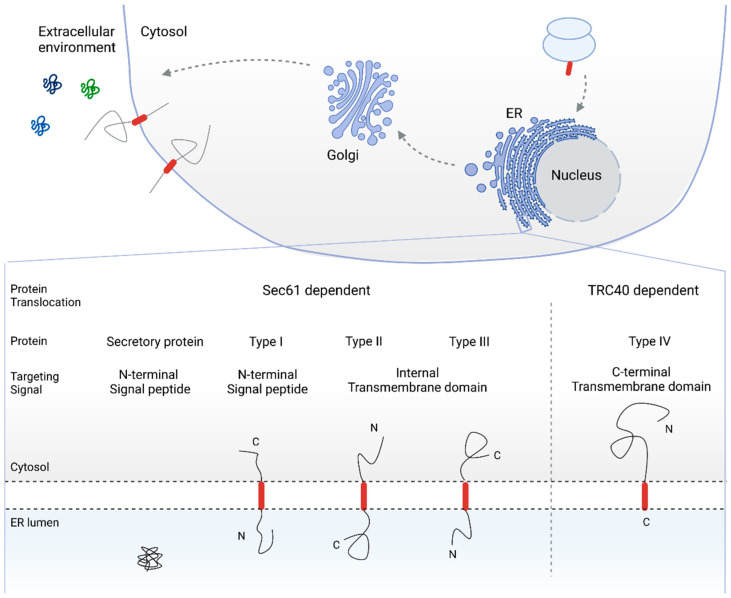
Overview of the secretory pathway for protein biogenesis and topology-based classification of secretory and single pass membrane proteins. Cleavable N-terminal SPs target secretory and type I membrane proteins to the ER membrane, resulting in an N-terminally translocated topology. In the case of type II and type III membrane proteins, the TMD functions as a targeting sequence. Depending on the overall hydrophobicity and charge of the topological sequences adjacent to the TMD, the C- or N-terminal end of the protein is translocated into the ER lumen (type II and type III single pass membrane protein, respectively). Type IV, or TA proteins, are targeted to the ER membrane via the C-terminal TMD. As a result, TA proteins are post-translationally translocated via the TRC-40 pathway. SP: signal peptide, ER: endoplasmic reticulum, TMD: transmembrane domain, TA: tail-anchored, TRC-40: transmembrane recognition complex subunit of 40 kDa.

With the evolution of simple cellular structures to multi organelle compartmentalized cells, the transport of proteins across biological membranes has become an unavoidable challenge. Extracellular and integral membrane proteins—synthesized in the cytosol—need to be translocated either across or integrated into bilipid membranes, in order to reach their final destination. Since the discovery of the secretory pathway [1,2,3,4,5], numerous studies have shed light on the different targeting signals, translocation modes, and pathways used by proteins to cross the endoplasmic reticulum (ER) membrane, which is the first and decisive step in the secretory pathway for protein biogenesis (see Figure 1) [6,7,8,9,10,11,12,13,14,15,16,17,18,19,20,21,22]. After maturation in the ER lumen, the proteins are embedded in vesicles and travel through the Golgi apparatus to the cell membrane. Here, the vesicles fuse with the cell membrane, resulting in the expression of the membrane protein at the cell surface or in the secretion of the soluble protein into the extracellular environment.

The targeting signals that drive proteins toward the secretory pathway include N-terminal (cleavable) signal peptides (SPs) as well as transmembrane domains (TMDs). As SPs and TMDs are intrinsic targeting signals for the Sec61 dependent pathway for protein co- and post-translational translocation [8,14,23,24,25,26,27], C-terminally located targeting signals route the respective protein to a different translocation pathway. In tail-anchored (TA) proteins, for instance, the TMD serves as the ER membrane targeting signal. The specific C-terminal location of the targeting TMD, however, restrict TA proteins to the transmembrane recognition complex subunit of the 40 kDa (TRC40) pathway for post-translational translocation as the targeting TMD emerges from the ribosome only when translation is completed [22,28,29,30,31,32,33,34]. In fact, single pass membrane proteins are often classified based on their targeting signal and topology after ER translocation (see Figure 1).

Evidenced by the evolutionary conservation of the translocation pathways over the different domains of life, correct translocation of proteins across the ER membrane is essential for the proper functioning of cells [21,35,36,37].

Small molecule inhibitors have therefore become an attractive tool to gain insights into the complex multistep nature of the different translocation routes known today [38,39,40,41,42]. However, new insights in this domain have quickly arisen. In this review, we update the previous knowledge of Sec61 dependent protein translocation in higher eukaryotes, and discuss the newest insights and mode of action of specific translocation inhibitors. Additional focus is placed on the interaction sites of each inhibitor within the translocation complex and screening methods to identify novel signal recognition particle (SRP)-Sec61 specific pathway inhibitors.

## 2. The Sec61 Dependent Pathway for Co- and Post-Translational Protein Translocation

**Figure 2:**
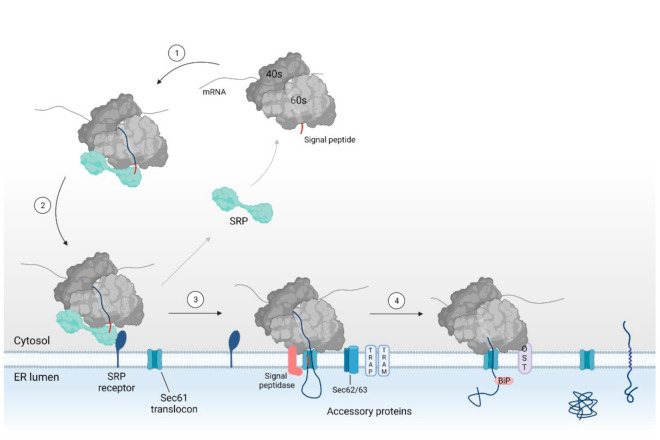
Overview of the SRP dependent pathway for co-translational translocation via the Sec61 translocon. A secretory or integral membrane protein is targeted toward the ER membrane by means of SRP binding to the signal sequence (i.e., the SP or TMD (steps 1–2)). SRP binding stalls protein translation to keep the nascent chain in a translocation competent state. At the ER membrane, SRP interacts with the SRP receptor. The RNC complex is then transferred to the Sec61 translocon (step 3). Interaction of the ribosome with the translocon reinitiates translation and induces conformational changes within Sec61α, eventually leading to protein translocation. In the case of a weak hydrophobic SP or TMD, the protein requires help from accessory proteins such as TRAP, TRAM, Sec62, and/or Sec63 for protein translocation. In the ER lumen, the SP is cleaved by the signal peptidase complex and the protein is glycosylated by the OST complex (step 4). SRP: signal recognition particle, ER: endoplasmic reticulum, SP: signal peptide, TMD: transmembrane domain, RNC: ribosomal nascent chain, TRAP: translocon-associated protein, TRAM: translocating chain-associating membrane protein, OST: oligosaccharyl transferase.

The Sec61 dependent translocation can occur in two modes (i.e., co- or post-translational translocation). Intrinsic to the terms, they refer to the translocation process occurring simultaneous with or after completion of protein translation, respectively. Post-translational translocation is most commonly used by small secretory proteins (SSP) (less than approximately 100 amino acid residues) and is best understood in fungi and bacteria. Co-translational translocation of proteins over the ER membrane, on the other hand, is a complex multistep process, as shown in Figure 2, which is mostly employed by higher eukaryotes [16,21,25,43,44,45].

In short, when the targeting signal emerges from the ribosome, it is recognized and bound to by SRP. Upon binding of SRP, protein translation is stalled and the ribosome-nascent chain (RNC) complex is targeted to the ER membrane through binding of SRP to its receptor [46,47,48,49,50]. Next, the targeting sequence interacts with the Sec61 translocon (i.e., the protein conducting channel). Binding of the ribosome to the translocon reinitiates translation of the nascent chain and induces translocation of the preprotein into the ER lumen [27,49,50,51,52,53,54]. In the lumen, the signal peptidase and oligosaccharyl transferase (OST) complex allow for further maturation of the translocated preprotein by cleaving the protein’s signal peptide and by glycosylation of the mature protein part, respectively [55,56,57,58,59].

SRP mediated protein targeting to the ER membrane is the most common in eukaryotes and therefore forms the focus of this review. However, proteins can also be SRP independently targeted to the ER membrane, in which case specific chaperone activity is required. For an overview of SRP-independent pathways for protein targeting to the ER, the reader is referred to other publications [11,12,16,43,60,61,62,63].

### 2.1. SRP Dependent Protein Targeting to the ER Membrane Keeps the Protein in a Translocation Competent State

When a secretory or integral membrane protein is translated in the cytosol and the targeting signal (i.e., SP or TMD) emerges from the ribosomal exit tunnel, it is recognized and bound to by SRP [46,47,64,65,66,67,68] (see Figure 2). SRP is a ribonucleoprotein complex consisting of six subunits (SRP9, 14, 19, 54, 68 and 72 m) and a 7S RNA molecule, which assemble into two SRP domains [22,46,48]. SRP 19, SRP54, SRP68, and SRP72 as well as the majority of the SRP RNA make up the S domain of SRP, which holds the recognition and binding site for the emerging SP. The remaining two proteins SRP9 and SRP14 as well as the 5′ and 3′ end of the RNA molecule form the Alu domain of SRP [46]. The Alu domain interacts with the ribosome elongation site, resulting in the transient retardation of protein translation [46,47,48]. Hence, SRP binding to the RNC complex locks the nascent chain in a translocation competent (i.e., unfolded) state by inducing a translational arrest.

Next, the RNC complex is targeted toward the ER membrane. Here, SRP interacts with the SRP receptor (SR) [46,47,64,65,66,67,68]. The SR then mediates the transfer of the RNC complex to the Sec61 translocon, the central component, and protein-conducting channel of the Sec61 dependent pathway for protein translocation [22,46,47,48].

### 2.2. Binding of the RNC Complex Induces Dynamic Conformational Changes in the Translocon

**Figure 3:**
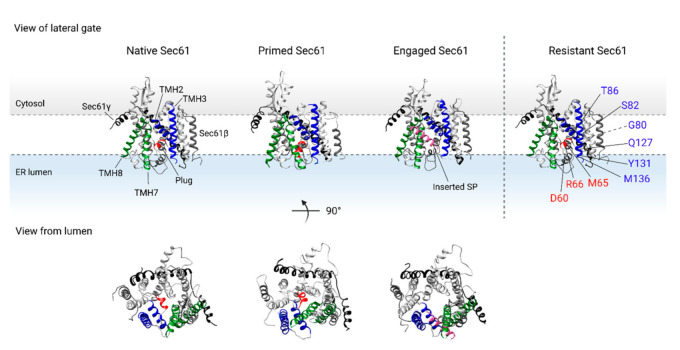
Dynamics of the TMHs of the Sec61 translocon (PDB 5A6U [69]) upon binding of the ribosome (primed state, PDB 3J7Q [52]) and insertion of the SP (engaged state, PDB 3JC2 [27]). Sec61α is shown in grey, Sec61β is shown in dark grey, and Sec61γ is shown in black. The interhelical interaction between TMH2 and TMH3 (shown in blue) on one half of the translocon, and TMH7 and TMH8 (shown in green) on the other half of the translocon form the lateral gate of Sec61α. Additionally, the translocon is closed axially by the lumenal plug domain of TMH2 (shown in red). Binding of the ribosome disrupts the interaction of TMH3 and TMH8 of the lateral gate and primes the translocon for insertion of the nascent protein chain, while the plug domain remains in place. The SP of the nascent chain (shown in pink) interacts with the lateral gate of the translocon, resulting in lateral escape from the translocon and insertion of the TMD into the ER membrane. In addition, the plug domain is displaced to allow for protein translocation into the ER lumen. Resistance conferring mutations located in the lateral gate or plug domain of Sec61α are shown in ‘Resistant Sec61’. TMH: transmembrane helix, SP: signal peptide, TMD: transmembrane domain, ER: endoplasmic reticulum.

The Sec61 translocon is a heterotrimeric complex that consists of Sec61α, β, and γ monomers (see Figure 3). The Sec61α subunit, composed of ten transmembrane helices (TMH), forms the central pore of the translocon [27,51,52,53,54,69,70]. In the quiescent, or native state, the translocon is axially closed by a lumenal plug domain in the central pore of the complex (see Figure 3, depicted as a single helix in red). In addition, the translocon is also laterally sealed by the lateral gate formed by the interhelical interactions between TMH2 and TMH3 (blue helices in Figure 3) and TMH7 and TMH8 (green helices in Figure 3) [51,52,53]. The interface between TMH2 and TMH7 near the cytosolic side of the translocon also serves as the recognition site for the targeting sequence of the protein nascent chain [27].

Structural studies have shown that binding of the RNC complex to the translocon triggers dynamic conformational changes within Sec61α, resulting in the interrupted interhelical contact between the lateral gate TMH3 and TMH8 (see Figure 3) ‘primed Sec61’ [35,55,56,57,58,59]. Interestingly, the position of the plug domain, which seals the translocon on the lumenal side of the ER membrane, is almost unaltered upon ribosome binding [51,70]. Hence, ribosome binding to the Sec61 translocon reinitiates protein translation by the release of SRP, and primes the translocon to accept an incoming nascent chain.

The inserting nascent chain can then interact with the recognition site in the lateral gate, which further opens the lateral gate, and displaces the plug domain so that the translocon is opened toward the lipid bilayer for TMD insertion, and toward the lumen for protein translocation [14,27,51,53,54,71].

### 2.3. Assisted Opening of the Sec61 Translocon

With the rise in structural models explaining the dynamic interactions of the Sec61 translocon upon protein insertion, it has become clear that the hydrophobic strength of the targeting signal is crucial for protein translocation. After all, the SP and/or TMD needs to be sufficiently hydrophobic to disrupt the interhelical hydrophobic interaction between the TMHs of the lateral gate to open the translocon for lateral escape into the ER membrane [27,51,52,69]. In addition, the SP and/or TMD need to displace the plug domain in order for the protein to translocate over the ER membrane.

Hence, proteins with a—so-called—weak hydrophobic SP and/or TMD require additional accessory components such as the translocon-associated protein (TRAP), translocating chain-associated membrane protein (TRAM), Sec62, and/or Sec63 for the translocation into the ER lumen. The specific accessory translocation machinery that is required, is thought to be protein, and thus SP/TMD specific [14,54,71,72,73,74,75,76,77,78,79].

### 2.4. Chaperone Mediated Completion of Protein Translocation and Post-Translational Modifications in the ER Lumen

For the translocation of the last amino acid residues that remain in the ribosomal exit tunnel when translation is completed, proteins rely on the binding immunoglobin protein (BiP), a lumenal translocation chaperone. BiP acts as a molecular ratchet by binding to the preprotein and pulling it toward the ER lumen to complete translocation in an ATP dependent manner [80,81,82,83]. Once translocated, the proteins are post-translationally modified in the ER lumen. For instance, the SP is cleaved from the preprotein by the signal peptidase complex and the preprotein is glycosylated by the oligosaccharyl-transferase (OST) complex [55,57,58,59].

## 3. Translocation Inhibitors of the Sec61 Dependent Protein Translocation Pathway

Being a multistep process, ER protein transport provides many pitfalls for protein mis-translocation that are mostly corrected by cellular control systems and specialized clean-up pathways such as ER associated protein degradation (ERAD) [84,85,86,87,88,89]. The correct translocation of proteins is crucial for the proper functioning of cells. In fact, inefficient protein translocation has been linked to many liver, kidney, and metabolic diseases [36,90]. Cancer cells, on the other hand, depend heavily on efficient protein translocation into the ER to support their fast growth. As such, correct protein translocation is key for many fast-growing cancers [37,91,92,93,94,95,96,97]. In addition, viruses exploit the host ER protein translocation machinery for the synthesis of viral proteins and host related entry receptors [98,99,100,101,102,103]. It is therefore no surprise that different inhibitors have been identified that interact with the Sec61 dependent protein translocation process.

The inhibitors known today are natural products and synthetic small molecules that inhibit Sec61 dependent protein translocation with differential substrate selectivity. Evidenced by the fact that many inhibitors originate from therapeutic screening programs, the Sec61 translocon forms a promising target for therapeutic intervention (e.g., for anticancer, immunosuppressive, and/or antiviral treatment).

In the following section, we present an overview of the Sec61 inhibitors of protein translocation known today, with a focus on the discovery, structure–activity relationship (SAR), therapeutic activity, and (putative) interaction sites within the Sec61 translocon.

### 3.1. Sec61 Inhibitors of Natural Origin

#### 3.1.1. HUN7293, CAM741, and Cotransin

Cell adhesion molecules play a critical role in the immune response by regulating leucocyte migration and cell-to-cell interaction at the site of inflammation. Therefore, the expression of cell adhesion molecules has become an interesting therapeutic target in a variety of inflammatory and autoimmune diseases that are characterized by the overexpression of cell adhesion molecules [104]. With this rationale, a screening program for the inhibition of cell adhesion molecule expression was set up and led to the identification of HUN-7293 [105]. HUN-7293 is a fungal cyclic heptadepsipeptide that selectively inhibits the expression of three cell adhesion molecules (i.e., vascular cell adhesion molecule 1 (VCAM-1), intracellular adhesion molecule 1 (ICAM-1), and E-selectin) [104,105]. With the HUN-7293 compound as the lead molecule of this new class of therapeutic agents, a complete library of analogs was synthesized to study the SAR [105,106,107]. This led to the identification of new inhibitors of cell adhesion molecule expression that have eventually also paved the way to study Sec61 dependent protein translocation [105,108,109,110].

A first HUN7293 analog is CAM741, a cyclopeptolide that selectively inhibits the expression of VCAM-1 by inhibition of the VCAM-1 co-translational translocation in the ER lumen [111,112]. By means of chemical cross-linking experiments, the authors showed that in the presence of CAM741, the VCAM-1 SP adopts an altered positioning relative to the Sec61α subunit of the translocon [108]. Later, vascular endothelial growth factor (VEGF) was identified as a second substrate for CAM741 [113]. As seen for VCAM-1, the VEGF SP is also diverted to a different position within Sec61α [113]. Hence, it was suggested that CAM741 interferes with the interaction between the SP and the SP recognition site in the lateral gate of the translocon, resulting in the incorrect insertion of the nascent chain and subsequent inhibition of protein translocation.

Around the same time as the identification of CAM741, another HUN-7293 analog, cotransin, was identified [109]. Cotransin selectively inhibits the expression of VCAM-1 and p-selectin via the inhibition of the co-translational translocation of these proteins across the ER membrane. In parallel to the CAM741 study, Garrison et al. showed that the orientation of the VCAM-1 nascent chain, with regard to the different translocon subunits, was altered in the presence of cotransin [109]. Photoaffinity labelling of cotransins confirmed earlier experiments that were performed on minimal liposomes (containing only the components that are crucial for translocation, i.e., Sec61 and SR), namely that the Sec61α subunit serves as target site for cotransin activity [109]. Later, a small subset of secretory and membrane proteins were identified as additional substrates for cotransin. Among these were angiotensinogen, β-lactamase, corticotropin releasing factor 1, endothelin B receptor, and aquaporin 2 (see Table 1) [105,109,110,114,115,116]. Initially, researchers believed that cotransin acts in a SP discriminatory manner, as so far, only secretory and type I membrane proteins with a SP targeting signal have been identified as cotransin substrates. CT08 and CT09, two cotransin analogs, however, showed activity against tumor necrosis factor α (TNFα), a single pass type II membrane protein with a non-cleavable TMD as a targeting signal [115]. From a proteomics study on cotrasin, Klein et al. concluded that the biosynthesis of almost all secreted proteins was cotransin-sensitive at a saturating concentration, whereas only a small subset of integral membrane proteins was affected at this concentration. Interestingly, for the integral membrane protein fraction, a conformational TMD consensus motif mediating cotransin sensitivity could be identified [110]. Hence, a cleavable SP is not a strict requirement for cotransin activity, leading to an unanticipated breadth of additional cotransin substrates.

Resistance studies on cotransin and analogs have shown that the lumenal region between the plug domain and lateral gate of the translocon serves as the active site of cotransin (see Table 1 and Figure 3) [115,116]. MacKinnon et al. showed that cotransin binding nearby the plug domain stabilizes the partially opened gate of Sec61α. In this model, the SP is prevented from entering the translocon, and TMD integration is hampered by blocking displacement of the plug domain [112,116,117].

Given the prominent role of VCAM-1, ICAM-1, and TNFα in the cellular immune response, HUN7293 as well as the related molecules CAM741 and cotransins might also be interesting as immunosuppressive agents [115,118]. A more recently identified cotransin substrate is the oncoprotein human epidermal growth factor receptor 3 (HER3), suggesting a potential anticancer activity for cotransin [119]. In addition, by blocking the Sec61 translocon with cotransin, researchers were able to show the importance of the translocon to support viral replication of the influenza A virus (IAV), the human immunodeficiency virus (HIV), and Dengue virus, implicating ER protein transport as a potential antiviral strategy [100].

#### 3.1.2. Decatransin

In contrast to the earlier described inhibitors, fungal cyclic decadepsipeptide decatransin inhibits protein translocation independent of the targeting sequence, and translocation mode, suggesting a broad-spectrum activity. Resistance profiling studies indicate that decatransin binds to Sec61α in a similar, yet distinct manner than cotransin (see Table 1) [120]. Interestingly, cotransin and decatransin also showed cross inhibitory activity with the prokaryotic SecYEG translocon [120].

#### 3.1.3. Apratoxin A and Coibamide A

Apratoxin A and Coibamide A are small molecules isolated from marine cyanobacteria that were originally investigated for their anticancer activity [121,122,123,124,125,126,127,128]. Natural products from marine organisms have a track record of antiproliferative activity in a variety of cancer cells that has led to the development of several clinical candidates [129]. Examples of such candidates from marine cyanobacteria are anti-tubulin agents, the cryptophycins, dolastatins 10 and 15, and curacin A [129,130]. Marine cyanobacteria have been shown to be an inexhaustible source of cytotoxic depsipeptides applicable to cancer research and potential pharmaceutical development [131,132].

Of the five naturally occurring apratoxins, apratoxin A exhibits the highest potency in various cancer cell lines, as the antiproliferative activity was found to be in the low nanomolar range. The antiproliferative activity was later assigned to the apratoxin A induced G1-phase cell cycle arrest and apoptosis [124]. Proteomics revealed that apratoxin A has a broad-spectrum activity as it reversibly downmodulates the expression of numerous ER resident proteins and cancer associated receptors via the inhibition of the co-translational translocation process [125]. Substrates of apratoxin A include gp130, c-MET, HER-2, PDGFR-β, insulin-like growth factor 1β, FGFR, and VEGFR2 [125]. The biological activity and structure have prompted researchers to study the total synthesis of apratoxins [125,133]. Hence, SAR studies have further investigated the selectivity profile of apratoxins, giving rise to apratoxin S4, a synthetic analog, with a more favorable cytotoxicity profile in vivo [121].

Based on the knowledge of other translocation inhibitors (i.e., CAM741 and cotransin), Sec61α was the suggested target candidate of apratoxins. In fact, via a radioactively labelled analog, the Sec61 complex was indeed identified as the molecular target of apratoxins [121]. A competitive binding assay with HUN7293 showed that apratoxins and HUN7293 likely have different binding sites within the translocon [121]. These results were confirmed by an additional study: mutagenesis and competitive photocrosslinking indicate that apratoxin A binds to the Sec61α lateral gate in a distinct manner as was seen for cotransins [122]. In fact, a mutagenesis study revealed that T86 and Y131, two residues located near the lumenal end of TMH2 and TMH3, respectively, are important for apratoxin A activity (see Table 1 and Figure 3).

A recent study suggests an antiviral potential of apratoxins, namely against the SARS-CoV-2 virus [134]. Since many of the apratoxin substrates are receptors that are validated targets for anticancer therapy [125], apratoxin A was thought to be the first anticancer agent to act through the mechanism of co-translational translocation inhibition. Around the same time, however, coibamide A has prompted scientists to investigate it for its unprecedented anticancer activity in vitro [127].

Coibamide A inhibits the migration, invasion, and cell cycle progression of glioblastoma cells [123] and has a broad-spectrum activity that shows substrate overlap with apratoxin A [128,135]. The anticancer activity of coibamide A was also shown in in vivo murine models, however, medicinal chemistry approaches are required to limit the observed dose induced toxicity. SAR analysis showed that the cyclization of the coibamide peptide is crucial for the biological activity, as two linear analogs no longer showed antiproliferative activity against glio- and neuroblastoma cancer cells [123].

By means of a photoaffinity labelled coibamide analog, researchers were able to identify the Sec61 translocon as the main target for coibamide A [135]. Later, resistance profiling suggested a distinct binding mode of coibamide A to Sec61α compared to the other known inhibitors [135]. In fact, the S71 residue that conferred coibamide A resistance upon mutation is located near the plug domain, and is shared only with decatransin, in contrast to the binding site of other inhibitors that are located in the area of the lateral gate (see Table 1 and Figure 3).

Interestingly, a recent study showed impaired autophagy to underly the anticancer activity of coibamide A [136].

#### 3.1.4. Mycolactone

Mycolactone is a virulence factor produced by the Mycobacterium ulcerans and is responsible for the pathogenesis of Buruli ulcers, predominantly seen in West Africa, Australia, Asia, and South America. The immunosuppressive effect caused by mycolactone upon infection of Mycobacterium ulcerans was later assigned to a broad-spectrum inhibition of Sec61 dependent co-translational translocation of secretory proteins that are important in the innate and adaptive immune response such as cytokines, chemokines, and homing receptors into the ER [137,138,139,140,141,142,143,144]. Mycolactone has a complex chemical structure consisting of a 12-membered lactone ring and two polyketide-derived chains that branch from the core in a north and south position [144]. In fact, SAR studies on mycolactone show that the northern chain of the structure is crucial for the biological activity of mycolactone [144].

Competitive binding assays with cotransin showed that mycolactone dose dependently competes with cotransin for binding to the Sec61 translocon. Resistance studies later confirmed the Sec61 translocon, specifically residues near the plug domain of the translocon as the binding partner of mycolactone [145,146]. As summarized in Table 1, these binding sites overlap with other Sec61 inhibitors, suggesting a shared mechanism of action.

A proteomics study conducted on T-cells later confirmed the broad-spectrum activity of mycolactone, as 52 proteins were significantly downregulated in the presence of mycolactone. In fact, mycolactone substrates predominantly consist of single pass type I and type II membrane proteins, containing either a SP or TMD to target the proteins to the ER membrane [138,145,147]. Hence, mycolactone is indiscriminatory for the targeting signal. Later, a more selective effect of mycolactone was seen on the expression of SSPs, which translocate in a post-translational manner. At this point, mycolactone was hypothesized to stabilize the closed conformation of the Sec61 translocon. SSPs with a sufficiently hydrophobic SP were hypothesized to overcome mycolactone activity because of the short nature of their mature protein. The large mature protein part of substrates that are co-translationally translocated, however, retains them in the translocon, independent of the strength of their SP.

This hypothesis, however, was questioned with the determination of the 3D structure of a mycolactone inhibited Sec61 translocon. To date, mycolactone is the only known inhibitor for which a high-resolution structure of the inhibited Sec61 translocon exists [148]. Here, Gérard et al. showed that the conformation of Sec61 in the presence of mycolactone is actually favorable for SP engagement as mycolactone binding induces conformational changes that open the cytoplasmic end of TMH2 and TMH3 of the translocon [148]. The broad-spectrum activity of mycolactone, however, implies that the compound occupies a site in the translocon that is important for SP or TMD binding. In fact, structural analysis confirmed the mycolactone binding site in the cytosolic entrance of the translocon, normally occupied by the SP. Mycolactone is therefore thought to prevent the SP mediated opening of the translocon and subsequent dislocation of the plug domain [148]. Strikingly, Sec61α mutations that confer resistance to mycolactone activity are not located within the mycolactone binding pocket of Sec61α (see Table 1 and Figure 3). These resistance mutations, in fact, induce conformational changes in Sec61α that reduce the formation of the mycolactone binding site near the cytoplasmic end of the translocon [148]. As a consequence, mycolactone binds less efficiently to the mutant Sec61 translocons and no longer inhibits protein translocation into the ER lumen.

#### 3.1.5. Ipomoeassin F

Ipomoeassin F (IpoF) is a natural plant derived resin glycoside cytotoxin that showed a high anticancer potency in different cell lines [149,150,151]. Later, IpoF was shown to be a non-selective inhibitor of protein secretion via the co-translational translocation process [151,152,153]. In vitro translocation assays showed that IpoF is specific for the inhibition of Sec61 dependent protein translocation as tail-anchored proteins, but also type III single pass membrane proteins were resistant to IpoF activity [151]. Furthermore, SAR studies showed that the ring size of the IpoF structure is correlated to the biological activity, as ring expansion enhanced IpoF cytotoxicity in cells and its potency to reduce in vitro protein translocation [154].

Resistance profiling shows that IpoF competes with other known inhibitors such as cotransin, apratoxin A, and mycolactone for Sec61α binding, suggesting that these inhibitors have at least partially overlapping interaction sites within the translocon [151].

In addition to the anticancer potency of IpoF, an in vitro SARS-CoV-2 antiviral activity was recently reported for IpoF through the inhibition of the co-translational translocation process of the SARS-CoV-2 spike proteins and the host cell membrane receptor ACE2 [152].

### 3.2. Synthetic Sec61 Inhibitors

#### 3.2.1. Cyclotriazadisulfonamide

In contrast to the previously discussed Sec61 inhibitors, cyclotriazadisulfonamide (CADA) is a synthetic small molecule translocation inhibitor that was first discovered during a human immunodeficiency virus (HIV)-screening program [103]. It was shown that CADA downmodulates huCD4 expression on a wide range of cells [99,102,103]. Since huCD4 is the main entry receptor for HIV, the reduced expression of huCD4 in the presence of CADA explains the observed antiviral effect of the compound [102,103,162]. In addition to the reported CD4-mediated antiviral effect for CADA, recently, a CD8^+^ T-cell mediated immunosuppressive effect was described that is related to the CADA-induced suppression of CD137 upregulation [158]. Furthermore, partial downmodulation of the sortilin protein by CADA [159] has recently been linked to a reduction in progranulin-induced breast cancer stem cell propagation [163], thus, suggesting an additional anticancer effect for CADA.

The relatively small size of CADA stimulated the synthesis of numerous analogs that could be implemented in SAR studies [102,163,164,165,166,167,168,169]. These SAR studies were all based on the biological effect of CADA analogs on the cellular expression of the huCD4 receptor, and structure optimization resulted in improved activity going from µM to the nM range [165]. An important condition for the preservation of activity is the closed 12-membered ring structure of the compound, given that open ring analogs did not exert any activity on huCD4 [166]. A first quantitative SAR study pointed to the importance of a relatively large, hydrophobic tail group for high impact on huCD4 [164]. In contrast to the symmetrical nature of the lead compound CADA, a subsequent SAR study revealed that unsymmetrical CADA analogs with two different side arms exerted the highest activity [168].

Mechanistic studies showed that CADA directly interacts with huCD4 SP and its reorientation within the Sec61 translocon during the co-translational translocation process of the human CD4 preprotein [170]. In fact, CADA was the first translocation inhibitor for which a direct binding to a SP was shown [170], which distinguishes it from the group of Sec61 translocon binding inhibitors described above. Specific residues in the vicinity of the hydrophobic h-region of the huCD4 SP were identified as being critical for the sensitivity to CADA [171]. Furthermore, a proteomics study on T-cells was performed and identified only five substrates for CADA (see Table 1), suggesting a selective nature of the compound [103,157,158,159]. Importantly, all substrates carried a cleavable SP as a targeting sequence, implicating that these proteins are Sec61 selective proteins for co-translational translocation. One can thus speculate that the common factor, Sec61α, is a target for CADA binding, however, the importance of direct interaction of CADA with the protein SP cannot be ruled out [170]. Evidence to confirm these hypotheses is awaited as well as the analysis of more potent CADA analogs on substrate selectivity and translocation inhibition.

#### 3.2.2. Eeyarestatin

The ER to cytosol degradation pathway for the disposal of misfolded proteins is an attractive target of intervention for diseases characterized by impaired protein degradation such as Alzheimer’s, Parkinson’s, prion, and Huntington’s disease [172,173,174]. It was in this regard that Eeyarestatin (ES) I and II, two structurally related chemical molecules, were identified from a library to screen for ERAD inhibitors [172,173]. ESI and ESII were shown to bind with the ER membrane bound p97 complex of ERAD, finally resulting in hampered deubiquitination of misfolded proteins, an essential step for proper proteasomal degradation [175]. As a result, misfolded proteins accumulate and rapidly induce ER stress [175,176]. However, it became clear that ESI and ESII also interfere at a step prior to proteasomal degradation. In fact, studies on ESR35, an ESI analog, showed a broad-spectrum inhibition of protein translocation [160]. Further analysis of the ES compounds suggests that ES targets a component in the Sec61 translocon and thereby sterically prevents the transfer of the RNC complex from the SRP targeting machinery to the Sec61 translocation machinery [160].

Since ES, and other inhibitors for that matter, interacts with the Sec61 translocon to prevent protein translocation into the ER lumen, they may indirectly induce Ca^2+^ leakage from the ER lumen, the major intracellular Ca^2+^ storage [177]. In fact, it was shown that ES, via its 5-NF moiety, induces Ca^2+^ leakage from cells [178,179]. ESI, ESII, and ES24, a minimal analog that closely resembles the 5-NF moiety of ES [179], have been shown to prevent protein translocation, however, while keeping Sec61 in a Ca^2+^ permeable state [178]. This apparent contradiction was reconciled in a mechanistic model for the action of ES compounds on Sec61 complexes [178]. Docking analysis of the Sec61 translocon structure revealed that ES, and particularly the 5-NF group, putatively interacts with the cytosolic end of the Sec61α lateral gate. The binding of ES in the space between TMH2 and TMH7 hampers conformational changes of Sec61α that are required for protein translocation [178]. Hence, this model suggests that ES binding stabilizes the primed, Ca^2+^ permeable, state of the Sec61 translocon by preventing the lateral gate to close [178].

Since the Sec61 translocon is tightly linked to the ERAD pathway for protein degradation, it is suggested that the inhibition of the ER translocation machinery might simultaneously also block both the retrotranslocation of misfolded proteins to the cytosol [160]. The resulting accumulation of cytosolic and misfolded proteins subsequently induces ER stress, which explains the cytotoxic effect of ESI in cellula, and even suggests an anticancer activity of ES [160,180].

In fact, induced tumor cell death upon ES treatment was reported in vitro, and appeared to be enhanced upon co-treatment with proteasomal inhibitors such as bortezomib [160,173,175,176,181,182,183]. Recently, an antibacterial activity of ES24, a smaller analog of ESI, was described. ES24 impairs protein translocation in E. coli via interaction with the SecYEG translocon, the prokaryotic orthologue of the Sec61 translocon [184].

#### 3.2.3. KZR-261 and KZR-834

The most recent Sec61 dependent protein translocation inhibitors are KZR-261 and KZR-834, two structural analogs that were identified in an anticancer medicinal chemistry screening program [161]. A proteomics study that assayed KZR-261 and KZR-834 activity on different tumor cell lines showed that both compounds had a broad-spectrum activity in vitro in the nanomolar range, with a preference for secreted and type I membrane proteins [161]. In fact, in vivo studies have been performed and have even led to the selection of KZR-261 for clinical development to profile the safety and early efficacy of this novel compound [161]. Among the other substrates are more therapeutic targets such as VEGF, VEGFR, and EGFR, suggesting, besides the anticancer activity, also an immunosuppressive potency for KZR-261 and KZR-834 [161].

## 4. High Throughput Screening Assays to Define Novel Inhibitors of the Sec61 Complex

Novel small molecule inhibitors of protein translocation at the Sec61 complex may be important innovations in pharmacology and may have several medicinal indications in the future. From the previous section, it is clear that Sec61 inhibitors act by a selective or non-selective mechanism of action [39,90].

Broad-spectrum or non-selective inhibitors impair translocation of many or even all proteins targeted to the Sec61 complex [39,90]. They may represent novel cancer drugs slowing down tumor cell growth. The advantage of Sec61 complex inhibitors over the still widely used cancer drugs affecting nucleic acid biosynthesis would be that they should not be mutagenic by themselves. Non-selective compounds may also be used to decelerate the production of viruses in infected cells, in particular, that of the enveloped viruses that possess integral membrane proteins at the surface using the Sec pathway. It is conceivable that such non-selective substances should target the Sec61 complex alone, rather than the signal sequences of the substrate proteins.

Selective inhibitors, instead, target translocation of a small subset of proteins at the Sec61 complex in a signal sequence discriminatory manner [39,90]. Selective inhibitors may be used in the future to downregulate the biosynthesis of proteins of interest. While a number of selective compounds have been described in the past decade, a specific substance inhibiting translocation of only one protein is not known thus far. Of note, the term selective inhibitor should only be used when proteomic experiments are performed, supporting this classification.

The inhibitors of translocation outlined above were described for the eukaryotic Sec61 complex present in the ER membrane. The orthologous bacterial SecYEG complex translocates proteins across the prokaryotic plasma membrane, therefore, inhibitors of the SecYEG complex may represent novel antibiotics that are urgently needed. A proof of principle for SecYEG inhibition was published, for example, decatransin [99] and eeyarestatin [81], although these compounds inhibit both the eukaryotic Sec61 complex and prokaryotic SecYEG.

The setup of high throughput screening assays for small molecule inhibitors of the Sec61 complex is notoriously difficult. This is essentially due to three properties of the Sec61 complex. (i) Most importantly, the heterotrimeric Sec61 core complex (Sec61αβγ) has no enzymatic activity by itself. Sec61α gating (i.e., the switch from the closed to the open state) is facilitated by ribosomes, signal sequences, and auxiliary factors such as TRAP and/or Sec62/Sec63 (recent reviews: [131,133]). Translocation itself is facilitated by the BiP chaperone ratchet mechanism at the ER lumenal side [83]. (ii) The accessibility of the complex for compounds in live cells is limited because it is only expressed intracellularly in the ER membrane. (iii) Finally, the complex is difficult to isolate and to reconstitute functionally in larger amounts, making in vitro assays unfavorable.

Although a lot of structural information has been published for the Sec61 complex in the past decade, no attempts have been made to define inhibitors by in silico screening. The main obstacle for in silico screening is the Sec61α architecture itself: it forms a huge and highly dynamic aqueous pore with diameters ranging from 12 to 22 Angstrom. The fact that the channel handles a multitude of different proteins implies that it has slightly different interaction sites for protein substrates. It is consequently very difficult to define specific inhibitor binding sites by in silico approaches.

**Figure 4:**
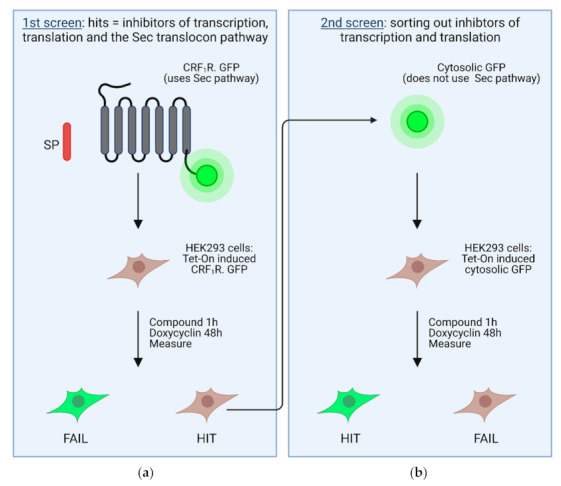
Scheme of the primary and the secondary screen in stably transfected HEK 293 cells. In the primary screen (**a**), Tet-On-controlled CRF1R.GFP was used, a GFP-tagged GPCR possessing a cleavable signal peptide that uses the SRP-Sec61 targeting/translocation pathway. The secondary screen (**b**) was performed with Tet-On-controlled, unfused, soluble GFP, which does not use the SRP-Sec61 targeting/translocation pathway. Hits of the primary screen were used in the secondary screen to deselect inhibitors of the transcription/translation machinery. Figure modified from [67].

Despite all of these potential obstacles, a whole-cell screening approach was recently published for inhibitors of the Sec61 complex using two succeeding screening steps [67]. In a primary screen, inhibitors for transcription, translation, and the SRP-Sec61 targeting/translocation pathway were selected (Figure 4a). To this end, a heptahelical G protein-coupled receptor was used as the target, which was C-terminally tagged with GPP (CRF1R.GFP; Figure 4a). The CRF1R.GFP possesses a cleavable signal peptide and thus uses the SRP-Sec61 targeting/translocation pathway [134]. SRP binding to the signal peptide of this construct should decelerate or even arrest translation. The idea is that inhibitors of the SRP-Sec61 targeting/translocation pathway from a compound library should decrease or even prevent CRF1R.GFP biosynthesis, and consequently expression of the C-terminal GFP tag, which could be measured fluorimetrically [77]. A decreased GFP fluorescence, however, may also be observed when inhibitors of the transcription/translation machinery are present. Taking all hit compounds of the primary screen, the latter were deselected with a secondary screen using unfused, cytosolic GFP protein as a target (Figure 4b) [77]. GFP alone does not use the SRP-Sec61 targeting/translocation pathway and its expression depends only on transcription and translation. Compounds were considered as inhibitors of the SRP-Sec61 targeting/translocation pathway when they behaved as hits in the primary screen, but not in the secondary screen [77].

Using this screening setup and a library of 37,312 substances, 1052 compounds were identified in the primary screen [77]. This number was reduced to 28 compounds following the secondary screen. Following an in vitro biosynthesis assay in live cells, five compounds were considered to represent real hits with a potential to inhibit the SRP-Sec61 targeting/translocation pathway. For one of them, namely FMP-401319-3, it could be shown by an in vitro transcription/translation/translocation assay that it acts indeed in a post targeting step at the level of the Sec61 complex. The potency of compound FMP-401319-3, however, was only in the low micromolar range. It remains to be determined whether it could be optimized by medicinal chemistry methods in the future. Using a much larger library might also be helpful to identify compounds with higher potency.

Of note, a slightly modified methodology may be used in the future to screen for substances affecting SecYEG, the bacterial ortholog of the Sec61 complex in order to derive novel antibiotic drugs. No such specific inhibitor for the prokaryotic SecYEG complex has been reported thus far, except for decatransin [120] and eeyarestatin [184], which both inhibit the Sec61 and SecYEG complexes.

## 5. Summary

Protein translocation is by far the most crucial process for the overall protein biogenesis and correct functioning of proteins in cellular processes, and homeostasis in general. This is evidenced by the fact that incorrect protein translocation is linked to numerous metabolic and protein folding diseases.

Today, different inhibitors of the Sec61 dependent protein translocation pathway have been identified. The chemical structure, compound concentration, and substrate targeting sequence are factors that ultimately contribute to the substrate specificity and selectivity of these compounds. As many inhibitors share binding regions within the Sec61α subunit, the translocon shows great potential as a molecular target in different therapeutic areas such as anticancer, immunosuppressive, and antiviral treatment. An intriguing fact of the inhibitors discussed in this review, is that they share a certain level of structural resemblance: they belong to the family of macrocyclic depsipeptides. The macrocyclic nature of the compounds, however, is associated with challenges regarding the synthesis, plasma stability, and/or stereochemical complexity.

By means of two-step whole cell screening approaches, researchers therefore aim to discover novel inhibitors specific to the SRP-Sec61 translocation pathway. A methodology that might also be expanded to screen for molecules that affect SecYEG, the bacterial ortholog of the Sec61 complex, in order to discover new antibiotic drugs. Undoubtedly, inhibitors of protein translocation will find their way into the clinic as promising therapeutics to treat various diseases.

## Figures and Tables

**Table 1:** Overview of Sec61 translocon inhibitors, substrate specificity, active concentration, and resistance conferring mutations.

Compound	Substrate Selectivity ^1^	Active Concentration ^2^	Sec61α Resistance Conferring Mutations	Reference
HUN-7293	VCAM-1, ICAM-1, E-selectin	IC_50_ 1–24 nM	Undefined	[104,107]
CAM741	VCAM-1, VEGF	IC_50_ 5–200 nM	Undefined	[105,108,111,113,118]
Cotransin	VCAM-1, P-selectin, Angiotensinogen, β-lactamase, CRF1, ET_B_R, AQP2, HER-3, TNF-α	IC_50_ 0.5–5 µM	R66, G80, S82, M136	[105,109,110,114,115,116,119]
Decatransin	Broad-spectrum	CC_50_ 30–40 nM	I41, D60, M65, R66, S71, G80, S82, M136	[120]
Apratoxin A	Broad-spectrum	CC_50_ 13 nM	T86, Y131	[122,155]
Coibamide A	Broad-spectrum	CC_50_ 10–100 nM	S71	[135,155,156]
Mycolactone	Broad-spectrum	IC_50_ 3–12 nM	R66, S71, G80, S82, T86, Q127, M136	[137,138,139,140,141,142,145]
Ipomoeassin F	Broad-spectrum	IC_50_ 50–120 µM ^3^	R66, S82	[151,152,153]
CADA	huCD4, SORT, CD137, DNAJC3, PTK7, ERLEC1	IC_50_ 0.2–2 µM	Undefined	[103,157,158,159]
Eeyarestatin	Broad-spectrum	IC_50_ 70–200 µM ^3^	Undefined	[160]
KZR-261/834	Broad-spectrum	IC_50_ nanomolar range	Undefined	[161]

^1^ VCAM-1: Vascular cell adhesion molecule 1, ICAM-1 Intercellular adhesion molecule 1, VEGF: Vascular endothelial growth factor, CRF1: Corticotropin releasing factor 1, ET_B_R: Endothelin B receptor, AQP2: Aquaporin 2, HER-3: Human epidermal growth factor receptor 3, TNF-α: Tumor necrosis factor α, huCD4: human cluster of differentiation 4, SORT: Sortilin, PTK7: Protein tyrosin kinase 7, ERLEC1: Endoplasmic reticulum lectin 1. ^2^ IC_50_: inhibitory concentration producing 50% reduction in biological activity. CC_50_: cytotoxic concentration causing 50% cell death. ^3^ IC_50_ in cell-free in vitro translocation assay.
